# Ferroptosis: a new approach for immunotherapy

**DOI:** 10.1038/s41420-020-00355-2

**Published:** 2020-11-12

**Authors:** Chong Zeng, Hongfeng Tang, Hanwen Chen, Mojuan Li, Dan Xiong

**Affiliations:** 1 Medical Research Center, Shunde Hospital, Southern Medical University (The First People’s Hospital of Shunde), Jiazi Road No.1, Lunjiao Street., Foshan, 528308 Guangdong China; 2 Department of Dermatology, Shunde Hospital, Southern Medical University (The First People’s Hospital of Shunde), Jiazi Road No.1, Lunjiao Street., Foshan, 528308 Guangdong China; 3 Department of Anesthesiology, Shunde Hospital, Southern Medical University (The First People’s Hospital of Shunde), Jiazi Road No.1, Lunjiao Street., Foshan, 528308 Guangdong China; 4 Department of Obstetrics and Gynecology, Nanhai Hospital Affiliated to Southern Medical University, Foping Road No.40., Foshan, 528200 Guangdong China; 5 Departments of Hematology, Shunde Hospital, Southern Medical University (The First People’s Hospital of Shunde), Jiazi Road No.1, Lunjiao Street., Foshan, 528308 Guangdong China

**Keywords:** Cancer immunotherapy, Phosphoinositol signalling

CD8^+^ T cells have become the central focus of novel cancer therapeutics. A recent report by Wang et al. in *Nature* identified that activated CD8^+^ T cells could induce tumor cell ferroptosis through the generated interferon-γ, which attenuated the glutamate–cystine antiporter system xc- and contributed to lipid peroxidation and ferroptosis. This novel observation suggested that CD8^+^ T cells initiated the tumor cell’s ferroptosis.

Ferroptosis is an iron-dependent form of cell death that can be driven by iron-dependent lipid ROS or small molecules, which is distinct from other forms of cell death. For instance, apoptosis, necrosis, autophagy, and pyroptosis have been identified by Dixon et al.^1^. Mechanistically, the ferroptosis is triggered by suppressing the central regulator, glutathione peroxidase 4 (GPX4), which is the direct inhibition of GPX4 activity or promotes its degradation with inducers. On the other hand, it indirectly inhibits GPX4 through blocking system Xc, which is a glutamate/cystine antiporter that exports intracellular glutamate in exchange for extracellular cystine to generate glutathione^2^. Ferroptosis is associated with tremendous diseases, such as degenerative diseases, kidney degeneration, and cancer^2^. Now, we read with an interesting research article published in *Nature* by Wang et al.^3^, who demonstrate that CD8^+^ T cells play a critical role in cancer immunotherapy through ferroptosis.

The paper by Wang and colleagues beautifully demonstrated that the central role of immunotherapy-activated CD8^+^ T cells promoted ferroptosis-specific lipid peroxidation and increased the antitumor efficacy of immunotherapy for the first time. They found that the activated CD8^+^ T-cell coculture with tumor cells would enhance lipid ROS and promote tumor cell ferroptosis, as well as the supernatant from activated CD8^+^ T cells^3^. As we know, interferon-γ (IFN-γ) and tumor necrosis factor (TNF) are secreted by activated CD8^+^ T cells. The anti-IFN-γ antibodies, anti-TNF antibodies, and CRISPR technology were performed and confirmed that IFN-γ was the primary factor that mediated tumor cell’s ferroptosis. Moreover, RNA sequencing analysis to identify *SLC7A11* and *SLC3A2* is strongly positively correlated with resistance to ferroptosis. Intriguingly, these genes consist of system Xc that is essential to ferroptosis. IFN-γ is able to increase interferon-regulatory factor 1 (IRF1) and inhibits both SLC7A11 and SLC3A2 expression. This will influence the system Xc, resulting in tumor cell ferroptosis via the Janus kinase (JAK) signal transducer and activator of the transcription 1 (STAT1) signaling pathway in tumor cells. The research represents a breakthrough in the field of tumor immunotherapy.

Antitumor immunotherapy has achieved great progress in the past few decades. On the one hand, adoptive cellular therapy enhanced robust expansion of tumor-infiltrating lymphocytes that boost immune-mediating antitumor response through manipulation of T cells. The application of chimeric antigen receptor (CAR) T-cell therapy in eliminating hematologic malignancies has acquired numerous success^4^. On the other hand, the immune-checkpoint blockade has revolutionized cancer treatment that alters T-cell function with a monoclonal antibody (mAb). Immune cell-targeted therapy, such as anti-CTLA-4, anti-PD-1, and anti-PD-L1, is currently among the most promising agent in clinical oncology. However, in this issue of *Nature*, new analyses presented by Wang et al. provides an integrated perspective of how the CD8^+^ T cells exert antitumor through ferroptosis and mediate the tumor cell’s death.

**Fig. 1:**
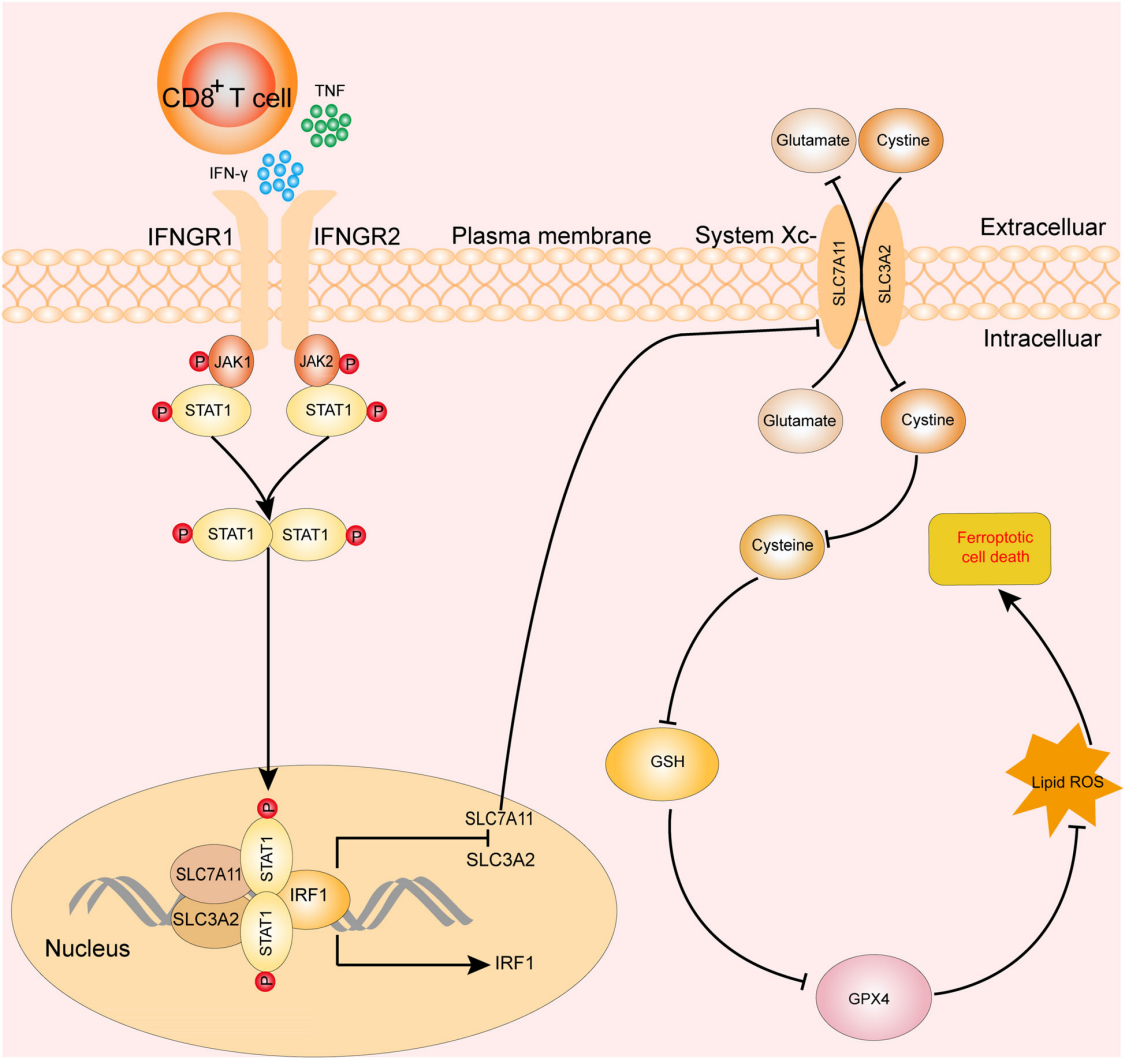
Mechanism of activated CD8^+^ T cells drives ferroptosis in tumor cells. The activated CD8^+^ T cells generate IFN-γ and TNF; afterward, IFN-γ binds to the extracellular domain IFN-gamma R1/R2, results in Jak1 and Jak2 activation, and then contributes to STAT1 phosphorylation. After STAT1 phosphorylation, its homodimerization and nuclear translocation promote IRF1; both SLC7A11 and SLC3A2 are inhibited. Subsequently, the glutamate–cystine antiporter system xc is impaired, eventually resulting in tumor cell ferroptosis.

CD8^+^ T cells play a central role in antitumor immunity, but their activity is inhibited in the tumor micronvironment^5^. Interestingly, a previous study has demonstrated that tumor and the infiltrating tumor-specific T cells interact with an adenovirus vaccine, can activate CD8^+^ T cells, and exert immunotherapy instigating in the tumor cells^6^. Dissimilarly, Yang et al. demonstrated that cholesterol metabolism was able to enhance mouse CD8^+^ T-cell immune response in cancer immunotherapy^7^. On the aspect of mechanism, Wang et al. illustrated that the activated CD8^+^ T cells release cytokine IFN-γ rather than TNF exerting an essential role in antitumors. The latest study by Williams et al.^8^ shows that in vitro CRISPR screen revealed that tumor cell-intrinsic IFN-γ signaling was necessary for optimal T-cell-mediated antitumor cell. This finding further supports the fundamentality of the IFN-γ signaling pathway in immunotherapy. Interestingly, the activated IFN-γ signaling pathway was capable of upregulating the IRF1, reducing the expression of SLC7A11 and SLC3A2 that influenced the system Xc result in ferroptosis (Fig. 1).

These new findings not only advance our understanding of CD8^+^ T cells in tumor immunotherapy, but also unveil the new mechanism mediating tumor cell ferroptosis, and may contribute significantly toward developing therapeutic applications. The emergence of CD8^+^ T cells mediates tumor cell death via ferroptosis as a novelty that conceptually also raises new interesting questions. The immune system and other immune cells or their subset also triggers ferroptosis in tumor immunotherapy. It deserves to be explored in the future. Therefore, we reasonably anticipate that critical target cells or molecules will become potential candidates for immunotherapy.
